# The Effects of High-Intensity Interval Training vs. Moderate-Intensity Continuous Training on Inflammatory Markers, Body Composition, and Physical Fitness in Overweight/Obese Survivors of Breast Cancer: A Randomized Controlled Clinical Trial

**DOI:** 10.3390/cancers13174386

**Published:** 2021-08-30

**Authors:** Babak Hooshmand Moghadam, Fateme Golestani, Reza Bagheri, Neda Cheraghloo, Mozhgan Eskandari, Alexei Wong, Michael Nordvall, Katsuhiko Suzuki, Parisa Pournemati

**Affiliations:** 1 Department of Exercise Physiology, University of Tehran, Tehran 1961733114, Iran; babak.hooshmand@mail.um.ac.ir; 2 Department of Exercise Physiology, Ferdowsi University of Mashhad, Mashhad 9177948974, Iran; 3 Department of Exercise Physiology, University of Birjand, Birjand 9717434765, Iran; fatemegolestani34@birjand.ac.ir (F.G.); Mozhgan.eskandari@birjand.ac.ir (M.E.); 4 Department of Exercise Physiology, University of Isfahan, Isfahan 8174673441, Iran; reza.bagheri@alumni.um.ac.ir; 5 Department of Epidemiology and Biostatistics, School of Public Health, Tehran University of Medical Sciences, Tehran 1417613151, Iran; n-cheraghloo@razi.tums.ac.ir; 6 Department of Health and Human Performance, Marymount University, Arlington, VA 22207, USA; awong@marymount.edu (A.W.); mnordval@marymount.edu (M.N.); 7 Faculty of Sport Sciences, Waseda University, 2-579-15 Mikajima, Tokorozawa 359-1192, Japan

**Keywords:** high-intensity intermittent exercise, moderate-intensity continuous exercise, adipokines, cytokines, breast cancer

## Abstract

**Simple Summary:**

We conducted this study to see whether HIIT and MICT can significantly decrease inflammatory markers through improving body composition in BCS. As such, these practical studies have a high degree of utility for the readership of this journal since most cancer survivors are afraid to perform physical activities. Although HIIT and MICT are effective strategies for improving inflammation, body composition, and physical fitness in BCS, our findings suggest that HIIT is superior to MICT in attenuating TNF-α and leptin as well as improving BM, FM, and LBS.

**Abstract:**

***Background***: Chronic inflammation associated with breast cancer (BC) poses a major challenge in care management and may be ameliorated by physical activity. This randomized controlled trial assessed the effects of a 12-week high-intensity interval training (HIIT) and moderate-intensity continuous training (MICT) on inflammatory markers, body composition, and physical fitness in BC survivors (BCS). ***Methods***: Forty BCS (age = 57 ± 1 years; body mass [BM] = 74.8 ± 1.5 kg; VO_2peak_ = 20.8 ± 2.1 mL·kg^−1^·min^−1^) were randomly assigned to three groups: HIIT (*n* = 15), MICT (*n* = 15), or control (CON; *n* = 15). The intervention groups (HIIT and MICT) performed their respective exercise protocols on a cycle ergometer 3 days/week for 12 weeks while the CON group maintained their current lifestyle. Baseline and post-intervention assessments included body composition (BM, fat mass (FM), lean mass (LM)), physical fitness (VO_2peak_, lower body strength (LBS), upper body strength (UBS)), and serum concentrations of tumor necrosis factor-alpha (TNF-α), interleukin-6 (IL-6), interleukin-8 (IL-8), interleukin-10 (IL-10), leptin, and adiponectin. ***Results***: Both intervention groups significantly (*p* < 0.05) decreased BM (HIIT = −1.8 kg, MICT = −0.91 kg), FM (HIIT = −0.81 kg, MICT = −0.18 kg), TNF-α (HIIT = −1.84 pg/mL, MICT = −0.99 pg/mL), IL-6 (HIIT = −0.71 pg/mL, MICT = −0.36 pg/mL), leptin (HIIT = −0.35 pg/mL, MICT = −0.16 pg/mL) and increased VO_2peak_ (HIIT = 0.95 mL·kg^−1^·min^−1^, MICT = 0.67 mL·kg^−1^·min^−1^), LBS (HIIT = 2.84 kg, MICT = 1.53 kg), UBS (HIIT = 0.53 kg, MICT = 0.53 kg), IL-10 (HIIT = 0.63 pg/mL, MICT = 0.38 pg/mL), and adiponectin (HIIT = 0.23 ng/mL, MICT = 0.1 ng/mL) compared to baseline. The changes in BM, FM, TNF-α, leptin, and LBS were significantly greater in HIIT compared to all other groups. ***Conclusions***: Our findings indicate that compared to the often-recommended MICT, HIIT may be a more beneficial exercise therapy for the improvement of inflammation, body composition and LBS in BCS; and consequently, merits long-term study

## 1. Introduction

Breast cancer (BC) is the most common cancer-related illness in women and is behind only lung cancer in overall mortality rate [1] Obesity is one of the main modifiers in cancer progression through chronic and low-grade inflammation, such that the risk of BC in obese patients is twice that of non-obese cancer patients [2,3]. Excess adipose tissue may enhance neoplasia and tumor progression in BC cells via mechanisms of chronic inflammation such as elevated pro-inflammatory cytokines including tumor necrosis factor-alpha (TNF-α), interleukin-6 (IL-6), interleukin-8 (IL-8), and decreased anti-inflammatory markers such as interleukin-10 (IL-10) [2,4,5]. Although cytokines were initially described as proteins that mediate and regulate the immune system and inflammatory processes, it is clear that TNF-a, IL-6, and IL-8 are involved in many other biological processes, including BC [6]. TNF-α, IL-6, and IL-8 are particularly characteristic tumorigenic cytokines involved at the onset and all subsequent stages of tumor development, including promotion, progression, and metastasis [6]. Furthermore, BC is characterized by increased production of pro-inflammatory adipokines such as leptin and reduced secretion of adiponectin, which itself has anti-inflammatory effects on macrophage activation and proliferation and is inversely correlated with adiposity [2]. Regular physical activity plays a pivotal role in cancer prevention by lowering concentrations of inflammatory markers such as cytokines [7].

Prior research has demonstrated a strong correlation between increased cardiorespiratory fitness (VO2_peak_) and reduced cancer-related mortality [8]. Although current guidelines for cancer survivors recommend the performance of moderate-intensity aerobic exercise [9,10], recent evidence indicates that high-intensity interval training (HIIT) more effectively improves VO_2peak_ compared to moderate-intensity continuous training (MICT) in BC survivors (BCS) [11]. This dose-response relationship was illustrated well through the work of Jones et al. [12], where MICT was unable to elicit significant alterations in plasma concentrations of IL-6, CRP, or TNF-α in BCS. Subsequent and limited investigations of populations with moderate to high concentrations of inflammatory markers, such as patients with BC, suggest that HIIT involving aerobic exercise, in particular, improves circulating concentrations of inflammatory markers [2]. However, to the best of our knowledge, no study has compared the effects of HIIT vs. MICT on inflammatory cytokines (IL-6, IL-8, IL-10, and TNF-α) and inflammatory adipokines (leptin and adiponectin) in BCS. Therefore, we conducted a randomized controlled trial design to investigate the role of HIIT vs. MICT in postmenopausal BCS on cytokines (IL-6, IL-8, IL-10, and TNF-α) and adipokines (leptin and adiponectin). In addition, we evaluated the effects of HIIT vs. MICT on body composition and physical fitness outcomes as related to inflammatory markers in the same study participants. We hypothesized that the HIIT intervention would elicit greater beneficial effects on inflammatory markers, body composition, and physical fitness outcomes compared to the MICT (or control) group.

## 2. Materials and Methods

### 2.1. Participants

**Figure 1:**
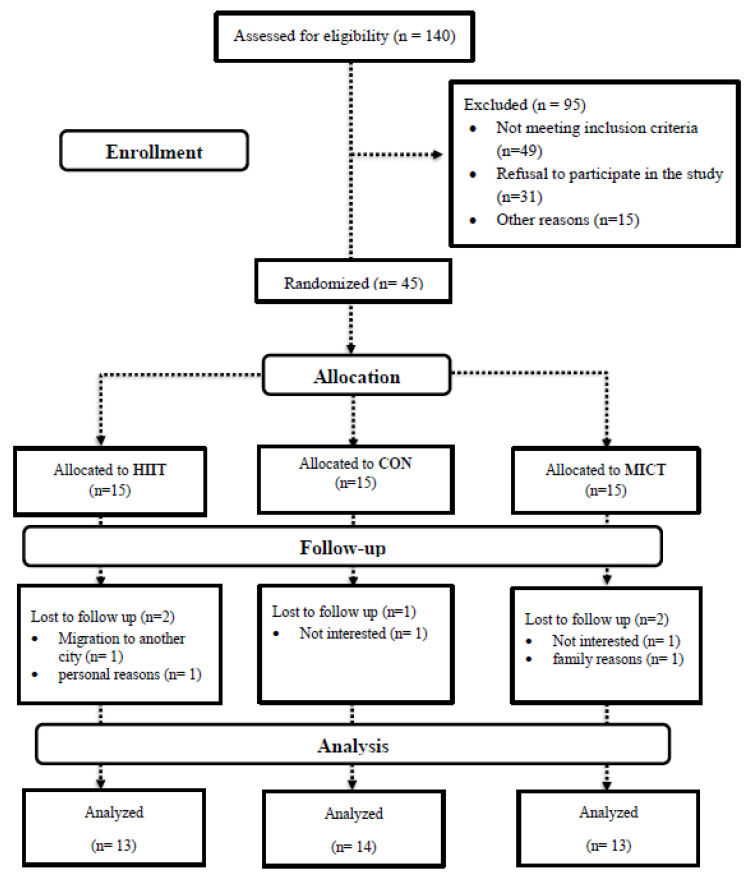
Flowchart of participants. **Abbreviations**: **HIIT**, high intensity interval training; **MICT**, moderate-intensity continuous training; **CON**, control.

Forty-five females (age 57 ± 1.0 years; height: 163.1 ± 6.7 cm; BMI (body mass index): 28.2 ± 2.2 kg·m^2^) having survived BC, and with the permission of their treating physician, voluntarily participated in this study. A flow chart of study participation and group allocation is illustrated in Figure 1. In addition, participant inclusion criteria were: age 50–75 years, post-menopausal (12 months elapsed since last menstruation period), BCS, cancer stage I, II, or III at the time of diagnosis, and outside completion of cancer course treatment of at least six months (includes completed surgery (mastectomy and lumpectomy), chemotherapy, radiation therapy) [2], sedentary (<60 min of physical activity per week), BMI ≥ 25 kg/m^2^ and body fat percentage (BFP) >30%, and ability to perform exercise training following approval by an oncologist and cardiologist. Exclusion criteria included a history of the central nervous system or bone metastatic cancers, any secondary cancers, cardiovascular disease, diabetes, hypertension, thyroid diseases, mental illness, infection, hormonal or immune disorders, smoking and consuming nutritional supplements, vitamins or alcohol. A physician evaluated participants’ history against the study criteria based on the American College of Sports Medicine (ACSM) guidelines [13] via utilization of the Physical Activity Readiness-Questionnaire (PAR-Q) and medical health/history questionnaire. In addition, participants were examined by a physician for mobility and other physical limitations as well as heart and respiratory conditions. Potential study participants were removed from consideration upon meeting any of the above exclusion criteria or physical limitations preventing exercise participation. Participant cancer-related information was obtained from patient medical records and is presented in Table 1. Prior to data collection, all study experimental procedures, benefits, and risks were explained to participants, who then provided written informed consent. This study was approved by the Ethics Committee of the Iranian Institute of Physical Education and Sports Sciences (IR.SSRI.REC.1398.055) and all experimental procedures were conducted following the Declaration of Helsinki. This study has been registered with the Iranian Registry of Clinical Trials (IRCT20190731044398N2).

### 2.2. Study Design

This study was a three-arm randomized controlled trial. Participants were familiarized with all testing procedures prior to baseline measurements. Following baseline measurements, participants were randomly assigned to one of three groups: high-intensity interval training (HIIT; *n* = 15), moderate-intensity continuous training (MICT; *n* = 15), or control (CON; *n* = 15). Participant group allocation was stratified using a commercially available online tool (www.randomizer.org, accessed on 8 July 2021). The HIIT and MICT group participants performed supervised exercise on a cycle ergometer three days/week for 12 weeks. Participants in the CON group were instructed to maintain their normal daily lifestyle, including normal self-care routines such as habitual diet and medication. All participants reported to an environmentally controlled laboratory twice to complete the pre (baseline) and post- outcome assessments (approximately 48 h after the last training session in the HIIT and MICT groups). All participants were instructed to report to the laboratory for testing in a normally hydrated state after an overnight fast. Moreover, the participants were also directed to avoid strenuous physical activity 48 h prior to each assessment period.

### 2.3. Anthropometrics and Body Composition Assessments

Upon arrival in the laboratory, participants were instructed to empty their bladders immediately before measurements. Body mass (BM) was measured using a digital scale (SECA, Germany) to the nearest 0.1 kg, and height was measured with a stadiometer (SECA, Germany) to the nearest 0.1 cm. BMI (by dividing BM in kg into the height in m^2^), fat mass (FM), and lean mass (LM) were evaluated by a multi-frequency bioelectrical impedance device (Inbody 770, Seoul, Korea) as previously described [14]. The test-retest reliability of the bioelectrical impedance was high (R = 0.95 to 0.99).

### 2.4. Blood Collection and Analysis

Fasting blood samples (~10 mL) were collected from the antecubital vein ~48 h before and after the last training session in the HIIT and MICT groups and at similar times in CON. Following the completion of blood sampling, samples were centrifuged at 3000 rpm for 10 min, and serum was stored at −80 °C until further analysis. Serum IL-8 (kit: R&D Co, sensitivity: 7.5 pg/mL), IL-10 (kit: R&D Co, sensitivity: 3.9 pg/mL), IL-6 (kit: Cusabio Co, sensitivity: 32.453 pg/mL), TNF-α (kit: Cusabio Co, sensitivity: 1.59 pg/mL), leptin (kit: Cusabio Co, sensitivity: 0.060 ng/mL), and adiponectin (kit: Cusabio Co, sensitivity: 1.102 ng/mL) concentrations were measured by using commercially available human ELISA kits. The intra- and inter-assay coefficients for all measures were <8% and <10%, respectively.

### 2.5. Physical Fitness Assessment

Peak oxygen uptake (VO_2peak_) was assessed utilizing a maximal incremental exercise test conducted on a cycle ergometer (with a ramp protocol of 15 W/min) according to ACSM standards and based on a linear heart rate (HR) response to increased VO2 uptake, as previously described [15]. Respiratory gases were collected throughout the test and then analyzed by the TrueMax 2400 metabolic system (Parvo Medics, Salt Lake City, UT, USA). Upper body strength (UBS) and lower body strength (LBS) were assessed using a one-repetition maximum (1RM) for chest press and leg extension machines, respectively. Following a brief warm-up, participants were progressed towards the maximum weight able to be lifted one time (1RM) through a full range of motion. All 1RM assessments were achieved within 3 to 5 attempts as previously described [16].

### 2.6. Diet

In an attempt to minimize dietary variability, participants submitted 3-day (2 weekdays and 1 weekend) food records prior to and after weeks 6 and 12 of the intervention/study. Each food item was individually entered into Diet Analysis Plus version 10 (Cengage, Boston, MA, USA) where total energy consumption and the energy derived from proteins, fats, and carbohydrates were evaluated [17].

### 2.7. Exercise Intervention

The two intervention groups (HIIT and MICT) performed 20-30 min of supervised exercise on a cycle ergometer (Monark 894E Ergometer) three times per week for 12 weeks. Participants’ HR (Polar, Finland) and rating of perceived exertion (RPE; modified Borg 6–20 scale) were monitored continuously (5-min intervals) to determine exercise response during each training session. Specifically, the MICT group completed both a 5-min warm-up and cool-down period at 50% of a participants’ peak power and a 20-min conditioning period at an intensity eliciting 55–65% of a participants’ peak power. The participants’ peak power was determined using the maximal incremental cycle test prior to the 12-week intervention. Exercise intensity over the duration of the study was adjusted to ensure participants in the MICT group exercised within the prescribed range of 55-65% of peak power or the equivalent of between 9 and 13 on the modified Borg scale (6–20) as determined by the study investigators. The HIIT group similarly completed 5 min warm-up and cool-down periods at 50% peak power as determined by the pre-intervention maximal incremental cycle exercise test. Following the warm-up, participants initially (week 1) completed four-cycle ergometer exercise intervals of 30 s duration with 2 min of active recovery (light resistance at the self-selected pedaling rate) between each interval. The number of exercise intervals was increased by one each week until the target of seven intervals was achieved by week 4, which was then maintained for the duration of the intervention. Pedaling rate during each interval was maintained at a rate of between 95 and 115 revolutions per minute (RPM) to ensure consistency across intervals. Cycle ergometer resistance was adjusted as participants progressed during the study intervention to maintain pedaling rate within the 95–115 RPM range and in order to achieve 90% or above maximum target HR by the fourth interval. HR and RPE were monitored continuously during each interval and in recovery in order to monitor participants’ responses to the exercise and made adjustments as necessary. All exercise sessions were supervised by an accredited exercise physiologist with experience working with cancer survivors. The exercise program was prescribed according to the ACSM and American Cancer Society guidelines for cancer survivors [18,19,20] and based on prior research in BCS [21].

### 2.8. Statistical Analysis

**Figure 2:**
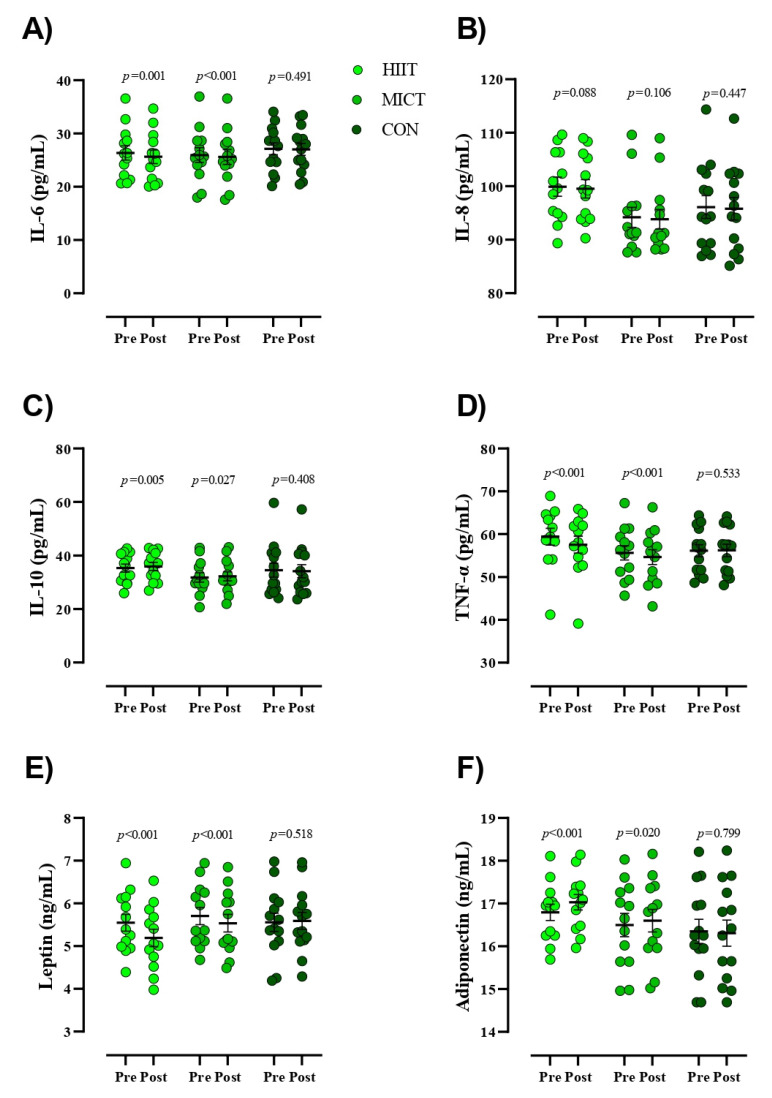
Serum concentrations of inflammatory markers from pre- to post-intervention. **Abbreviations**: **HIIT**, high intensity interval training; **MICT**, moderate-intensity continuous training; **CON**, control, (**A**), **IL-6**, interleukin-6; (**B**), **IL-8**, interleukin-8; (**C**), **IL-10**, interleukin-10; (**D**), **TNF-α**, tumor necrosis factor- α; (**E**), leptin; (**F**), adiponectin. Closed chartreuse indicates HIIT group, closed shamrock indicates MICT group, and closed pine indicates CON group. Error bars represent standard error of the mean.

**Figure 3:**
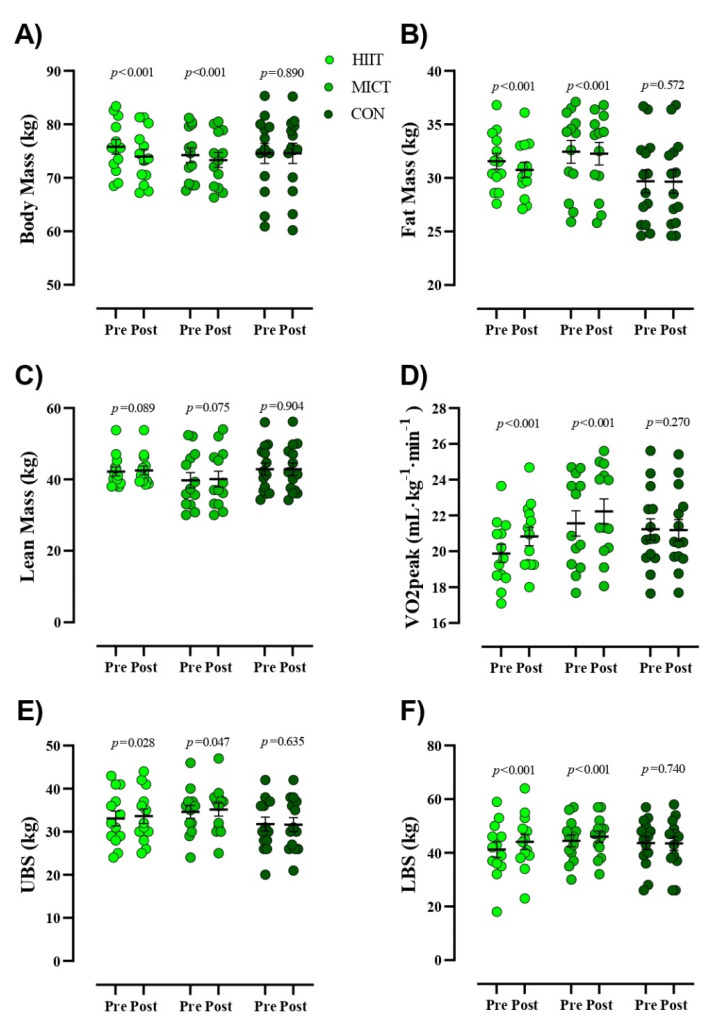
Body composition and physical fitness markers from pre- to post-intervention. (**A**) Body Mass (kg), (**B**) Fat Mass (kg), (**C**) Lean Mass (kg), (**D**) VO_2peak_ (mL·kg-1·min-1), (**E**) Upper body strength [UBS (kg)], (**F**) Lower body strength [LBS (kg)]. Closed chartreuse indicates HIIT group, closed shamrock indicates MICT group, and closed pine indicates CON group. Error bars represent standard error of the mean.

**Figure 4:**
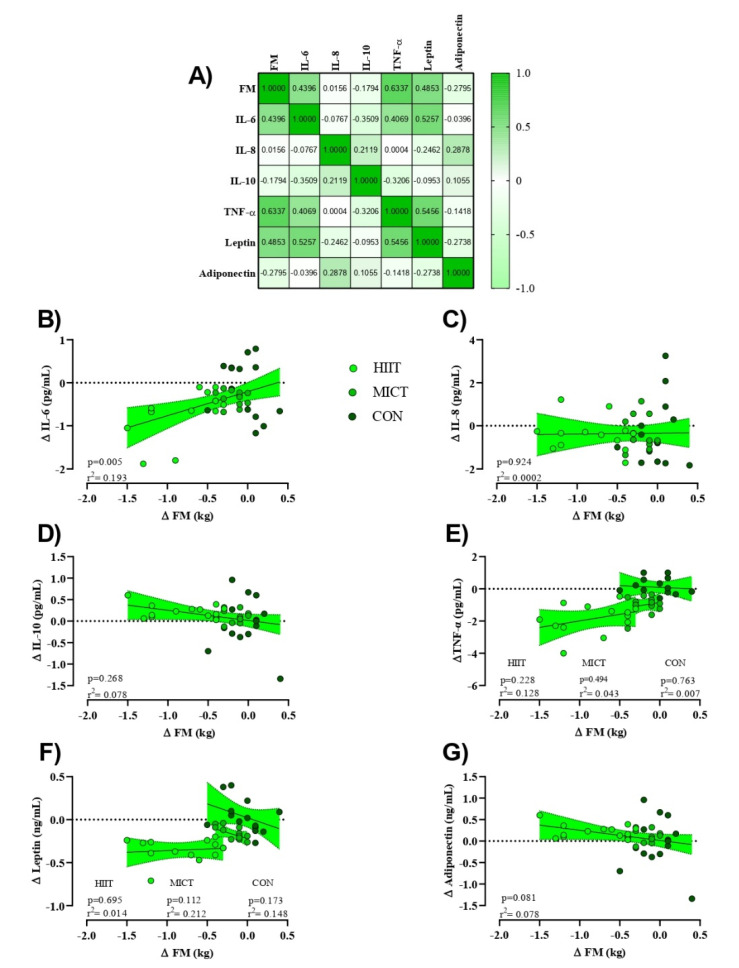
Relationships between change in FM (Δ FM [kg]) and changes in inflammatory markers (Δ marker). (**A**) Correlation matrix of Δ FM and inflammatory markers, r values as shown. Key indicates magnitude of r (green = −1 or 1, white = 0). (**B**–**G**) linear regression (Pearson’s) of Δ (marker) as a function of Δ FM (kg). Linear regression indicated by black line; 95% confidence intervals indicated by shaded chartreuse zones. Closed chartreuse indicates HIIT group, closed shamrock indicates MICT group, and closed pine indicates CON group.

Estimation of appropriate sample size was conducted using the G*Power analysis software. Our rationale for sample size was based on prior research that observed significant changes in cytokines (TNF-α, IL-6, and IL-10) following HIIT in BCS [22]. The analysis revealed that a minimum necessary sample size of 39 participants (13 per group) was needed to provide sufficient power (1- β) of 0.80 (α = 0.05) to detect significant changes in the concentration of these cytokines achieve a difference of 3–5% between the groups. However, we recruited 6 extra participants to allow for a 15% attrition rate, which was based on previous research [23]. The normality of data was confirmed using the Shapiro–Wilk test, and data are presented as mean ± standard deviation (SD). General between-group comparisons were performed using analysis of variance (ANOVA) with subsequent Tukey’s HSD test for determination of specific statistically significant group differences. We compared the means of two variables for the same subject using the paired t test. An analysis of covariance (ANCOVA) was performed to determine the mean between-group differences for each variable at baseline and at the conclusion of the study. Bonferroni tests were conducted to compare mean values between groups. Nutrition data was analyzed using repeated measures ANOVA analysis. The analysis was conducted using SPSS 26 (version 26, IBM-SPSS Inc., Chicago, IL, USA). *p* values less than 0.05 were considered statistically significant. Pearson’s linear regression was used to examine the relationship between continuous variables having an r^2^ value of >0.02, 0.13, and 0.26 as the threshold for a weak, moderate, and substantial effect, respectively, and as previously described [24]. In addition, data presented in Figure 2, Figure 3 and Figure 4 were prepared in GraphPad Prism software (Version 8.4.3, GraphPad Software, Inc., Jolla, CA, USA).

## 3. Results

### 3.1. Study Population

Between July 2019 and November 2019, we screened 140 BCS. After exclusion criteria were applied, 45 qualified for baseline evaluation and were subsequently randomized to either HIIT (*n* = 15), MICT (*n* = 15), or CON (*n* = 15) groups. Following randomization, five participants (two in both HIIT and MICT and one in the CON group) withdrew due to personal reasons (Figure 1). Data are presented for the 40 participants that successfully completed their 12-week intervention: 13 participants in both the HIIT and MICT groups and 14 in the CON group.

### 3.2. Dietary Intake, Side Effects, and Compliance with Intervention

There was no significant main effect of time nor group × time interactions for energy, carbohydrates, protein, and fat intakes over time (*p* > 0.05; Table 2). No adverse side effects were reported during the study. During the 12-week study period, exercise training adherence (% of training sessions completed) across the HIIT and MICT groups was 86.6%.

### 3.3. Inflammatory Markers

Figure 2 presents values for serum concentrations of inflammatory markers. Results of the present study indicated significant reductions within the intervention groups for serum concentrations of IL-6 [HIIT = −0.71 pg/mL (95% CI, −0.37 to −1), (*p* = 0.001) and MICT = −0.36 pg/mL (95% CI, −0.25 to −0.46), (*p* < 0.001)], TNF−α [HIIT = −1.84 pg/mL (95% CI, −1.26 to −2.41), (*p* < 0.001) and MICT = −0.99 pg/mL (95% CI, −0.65 to −1.32), (*p* < 0.001)], and leptin [HIIT = −0.35 ng/mL (95% CI, −0.27 to −0.43), (*p <* 0.001) and MICT = −0.16 ng/mL (95% CI, −0.11 to −0.22), (*p* < 0.001)] over time. Conversely, significant increases were observed within groups for serum concentrations of IL-10 [HIIT = 0.63 pg/mL (95% CI, 1 to 0.23), (*p =* 0.005) and MICT = 0.38 pg/mL (95% CI, 0.72 to 0.05), (*p* = 0.027)] and adiponectin [HIIT = 0.23 ng/mL (95% CI, 0.32 to 0.13), (*p <* 0.001) and MICT = 0.1 ng/mL (95% CI, 0.18 to 0.01), (*p* = 0.020)] over time. None of the observed alterations in serum inflammatory markers for the HIIT and MICT groups were observed in the CON group (*p* > 0.05). Additionally, there were no changes in serum concentrations of IL-8 within all groups pre to post study. ANCOVA revealed that decreases in post serum concentrations of IL-6 and increases in IL-10 were significantly greater in HIIT compared to CON (Table 3). Reductions in post serum concentrations of TNF-α and leptin in the HIIT group were significantly more pronounced compared to the MICT and CON groups.

### 3.4. Body Composition and Physical Fitness

Figure 3 presents values for body composition and physical fitness. Participants in both intervention groups had significantly reduced BM (HIIT = −1.8 kg (95% CI, −1.35 to −2.30), (*p* < 0.001) and MICT = −0.91 kg (95% CI, −0.64 to −1.18), (*p* < 0.001)) and FM (HIIT = −0.81 kg (95% CI, −0.56 to −1), (*p* < 0.001) and MICT = −0.18 kg (95% CI, −0.09 to −0.26), (*p <* 0.001)) over time, which was not noted in CON. No significant alterations in LM (*p* > 0.05) were observed in any of the groups in the present study. ANCOVA revealed that reductions in post-intervention BM and FM in HIIT participants were significantly more pronounced than all other groups (Table 3). Participants in both intervention groups significantly increased VO_2peak_ (HIIT = 0.95 mL·kg^−1^·min^−1^ (95% CI, 1.21 to 0.68), (*p <* 0.001) and MICT = 0.67 mL·kg^−1^·min^−1^ (95% CI, 0.85 to 0.50), (*p* < 0.001)), LBS (HIIT = 2.84 kg (95% CI, 3.53 to 2.15), (*p* < 0.001) and MICT = 1.53 kg (95% CI, 2.12 to 0.95), (*p* < 0.001)), and UBS (HIIT = 0.53 kg (95% CI, 1 to 0.06), (*p =* 0.028) and MICT = 0.53 kg (95% CI, 1 to 0.008), (*p* = 0.047)) over time, which was not noted in CON. ANCOVA revealed that increased post-intervention LBS values in the HIIT group were significantly more pronounced than all other groups, whereas no between group differences were noted for UBS (Table 3). In addition, while observed increases in Vo_2peak_ for both HIIT and MICT groups were significantly different (*p* < 0.001) from CON, there were no observed differences between exercise interventions.

### 3.5. Linear Regressions

To assess the potential relationships between training-induced changes in FM (Δ FM) on inflammatory markers (Δ (marker)) independent of HIIT or MICT interventions (e.g., pooled), linear regression analyses were used to generate a correlation matrix (Figure 4A). While serum concentrations of IL-10 and adiponectin showed a moderate albeit insignificant negative relationship with Δ FM (adiponectin showed a stronger relationship), IL-6, TNF-α, and leptin showed a statistically insignificant yet moderate positive relationship (TNF-α elicited a stronger relationship). Serum concentrations of IL-8 showed a weak positive relationship with Δ FM. Linear regression analyses for individual Δ marker as a function of Δ FM were first examined by utilizing extra sum-of-squares to determine if pooled data could be considered as a single model. Results indicated that all data except for serum concentrations of TNF-α and leptin could be considered as a single group. Subsequently, only serum concentrations of Δ IL-6 showed a significant direct relationship with training-induced changes in FM (*p* = 0.005), Figure 4B).

## 4. Discussion

This randomized controlled clinical trial attempted to evaluate whether 12 weeks of HIIT or MICT could improve inflammatory markers, body composition, and physical fitness in BCS. The main findings of our study were that both HIIT and MICT significantly decreased serum concentrations of TNF-α, IL-6, and leptin and increased serum concentrations of IL-10 and adiponectin. However, and indicating importance for achieving a necessary exercise intensity training stimulus for certain outcomes, the changes in serum concentrations of IL-6 and IL-10 were significantly greater in the HIIT group compared to the CON group (but not MICT vs. CON). Further, alterations in serum concentrations of TNF-α and leptin in the HIIT group were significantly greater compared to the other groups. Similarly, BM and FM significantly decreased, and LBS increased following 12 weeks of HIIT and MICT, with more pronounced changes noted in the HIIT group. Lastly, serum concentrations of Δ IL-6 showed a significant direct relationship with training-induced changes in FM.

### 4.1. Effects of HIIT and MICT on Inflammatory Markers and FM

We found a significant reduction in serum concentrations of TNF-α and IL-6 in both HIIT and MICT groups; however, the same was not observed in serum concentrations of IL-8. Further, serum concentrations of IL-10 in the present study significantly increased in both exercise interventions. Our findings are in accordance with prior research that noted that 16 weeks of resistance training induced alterations in serum concentrations of TNF-a in BCS [23]. Similarly, research by Serra et al. [25] reported significant reductions in plasma concentrations and adipose tissue levels of IL-6 and TNF-α and enhancement in IL-8 after 16 weeks of moderate-intensity whole-body resistance training in BCS. Utilizing a modified HIIT protocol compared to the present study, Alizadeh et al. [22] demonstrated that 12 weeks of HIIT led to a significant reduction in serum concentrations of TNF-α and IL-6 and a significant increase in IL-10 in BC patients. Our results suggest that both HIIT and MICT interventions provide sufficient exercise stimulus to decrease concentrations of certain pro-inflammatory markers in BCS. In recent years, large observational investigations on the suitability of HIIT for BCS have also emerged [8,26,27] since there is less known about the effects of HIIT compared to MICT in this population. Nevertheless, it appears that both HIIT and MICT may help regulate inflammation by inhibiting monocyte and macrophage infiltration into adipose tissue and phenotypic switching of macrophages also within adipose tissue [28]. Concurrent exercise-induced training increases in epinephrine concentrations may subsequently inhibit and thus decrease serum concentrations of TNF-α, IL-8, and IL-6 [29]. It is well understood that weight loss resulting from exercise training reduces adipocytes and consequently the number of macrophage and endothelial cells [30]. To this, increased BFP, in general, has been associated with heightened serum concentrations of TNF-α, IL-8, and IL-6 [22]. It appears in the present study that the enhanced FM lowering effect of HIIT proves of greater benefit in lowering certain pro-inflammatory markers in BCS. As such, observed reductions in serum concentrations of TNF-α and IL-6 seem dependent on FM and BM alterations in a linear manner, albeit only a significant relationship was noted for serum concentrations of Δ IL-6 and Δ FM. Overall, both HIIT and MICT appear to be effective and well-tolerated strategies for improving serum concentrations of TNF-α, IL-6, and IL-10 in BCS; however, compared to MICT, HIIT may be a more beneficial intervention for improving concentrations of these cytokines.

Leptin and adiponectin are secreted from adipose tissue and play a crucial role in energy intake and energy expenditure [31]. A growing body of research indicates that higher serum concentration of leptin and lower adiponectin are correlated with obesity and increased BC mortality rate [3,21,32,33]. Our study indicated decreased serum concentrations of leptin in both HIIT and MICT groups with greater alterations in the HIIT compared to the MICT group, while both HIIT and MICT groups observed similar and significant increases in serum concentrations of adiponectin. These results are contradictory to the findings of Swisher et al. [3], who reported an insignificant increase in serum adiponectin following 12 weeks of moderate-intensity aerobic exercise in women with BCS. However, Dieli-Conwright et al. [2] demonstrated a significant reduction in plasma concentrations of leptin and increased adiponectin after 16 weeks of a combined aerobic and resistance exercise training intervention in BCS. By acting on the central nervous system to reduce appetite and stimulate energy expenditure, leptin itself is regulated by exercise training via the sympathetic nervous system and α-adrenergic mechanisms to raise the oxidation of fatty acids in skeletal muscle by activation of AMP-activated protein kinase (AMPK) [34]. FM, along with leptin, significantly decreased in HIIT compared to the MICT group in the present study. Evidence suggests that serum concentrations of leptin are tightly correlated with BFP [35], a notion reinforced by the work of several authors who reported a positive association between reduced serum concentrations of leptin and decreased FM [33,34]. In the present study, and despite significant reductions in serum concentrations of leptin and FM in HIIT participants compared to MICT, no correlation between serum concentrations of leptin and FM existed, suggesting that reduced serum concentrations of leptin were independent of FM. Further research is needed to confirm these results.

### 4.2. Effects of HIIT and MICT on Physical Fitness and LM

Findings from the present study demonstrated significant improvements in VO_2peak_ occurred in both HIIT and MICT groups; however, HIIT provided no additional benefit compared to MICT on improving aerobic power. The literature remains divided on this topic such that improvements in VO_2peak_ may be independent of exercise training intensity in certain populations. For example, Wouda et al. [36] reported no significant between-group improvements in VO_2peak_ after 12 weeks of moderate- vs. high-intensity aerobic training in patients with incomplete spinal cord injury. In contrast to our results, Northey et al. [21] reported greater improvements in VO_2peak_ in a HIIT group following a modified program compared to ours versus MICT in women with BCS. Similarly, Toohey et al. [11] demonstrated a 19.3% increase in VO_2peak_ following just nine weeks of HIIT compared to a MICT intervention in BCS. Perhaps the exercise mode (cycling is a non-weight bearing activity involving lower body musculature rather exclusively), intensity (more total and frequent intervals, shorter rest periods, etc.), and duration (weeks of training) could explain the lack of difference between interventions in the present study; although it should be stressed that both training methods did improve aerobic capacity in this population. Different mechanisms may also underlie the improvements in VO_2peak_ in BCS dependent on exercise type and intensity and may be related to other physiological and metabolic mechanisms. Following HIIT, for example, increased VO_2peak_ has been associated with increased adenosine triphosphate (ATP) generation via phosphocreatine degradation and muscle glycogenolysis [37]. Furthermore, HIIT may induce greater improvements in vascular/endothelial function and cardiac output by enhanced cardiac contractility and increased muscle oxidative activity and capacity [37,38]. Nevertheless, and while observed in obese [37] and other populations [38], the relationship of these mechanisms to exercise-induced changes in aerobic capacity in BCS, in particular, remains unclear and warrants further investigation.

Data from the present study also indicated that HIIT participants experienced greater improvement in LBS as compared to MICT, results not dissimilar to Soriano-Maldonado et al. [39], who demonstrated that 12 weeks of combined resistance and aerobic exercise training significantly increased muscular strength (both upper and lower body) in BCS. Similarly, Serra et al. [25] showed that 16 weeks of resistance training improved muscular strength in postmenopausal BCS, which has been attributed to several neural adaptations such as increased number and frequency of motor unit recruitment [40]. The nervous system has a critical role in improving muscular strength and thus may help to explain the gains in muscular strength noted in the present study [41]. Because there were no significant changes in LM, the higher stimulus with HIIT (more forceful contractions and greater aerobic stimulus) may be related to enhanced neural adaptations and compared to MICT may explain the between-group differences in LBS observed in the present study.

### 4.3. Strengths and Limitations

The strengths of this investigation include the use of both HIIT and MICT protocols, which afforded direct comparisons between exercise training intensity and inflammatory and metabolic markers as well as fitness and body composition markers in overweight and obese BCS. Further strengths include the inclusion of sedentary women participants, strong HIIT and MICT compliance rates, and exercise protocols prescribed according to established guidelines and prior research [18,19,20,21]. Furthermore, valid and objective measures were used for the evaluation of inflammatory markers, body composition, and fitness. Nevertheless, this study was limited by a rather small number of participants in each group; however, sample size was evaluated, and our data often reached statistical significance. Additionally, bioelectrical impedance was utilized to measure body composition, an approach not as precise as dual-energy X-ray absorptiometry or hydrostatic weighing (gold standards in the assessment of body composition); however, prior research has shown BIA, when performed in a controlled environment, to be a valid and reliable method [42,43].

## 5. Conclusions

Although HIIT and MICT are effective strategies for improving inflammation, body composition, and physical fitness in BCS, our findings suggest that HIIT is superior to MICT in attenuating TNF-α and leptin as well as improving BM, FM, and LBS. Future research should address whether greater reductions in central adiposity in BCS through exercise training will demonstrate enhanced cytokine and adipokine regulation often associated with whole-body inflammation. We conclude, based on the results of this study, that BCS having recently completed treatment incorporate structured HIIT early in the survivorship continuum.

## Figures and Tables

**Table 1:** Information about the participants’ treatment.

		HIIT (*n* = 13)	MICT (*n* = 13)	CON (*n* = 14)	Total (*n* = 40)
**Cancer Stage**	I	4	3	4	11
	II	4	3	5	12
	III	5	7	5	17
**Treatment**	Surgery	2	3	3	8
	Surgery + chemotherapy	4	4	5	13
	Surgery + radiation	4	2	4	10
	Surgery + chemotherapy + radiation	3	4	2	9
**Hormonal therapy**	Tamoxifen	6	7	7	20
	aromatase inhibitors	5	4	5	14
	None	2	2	2	6

**Abbreviation: HIIT**, high intensity interval training group; **MICT**, moderate-intensity continuous training group; **CON**, control group.

**Table 2:** Energy and macronutrients at the before and at the end of week 6 and week 12.

Variables	Group	Baseline	6 Weeks	12 Weeks	*p* Value
**Energy (kcal/day)**	HIIT	1676.23 ± 54.91	1672.38 ± 51.13	1681.76 ± 46.87	0.793
	MICT	1671.23 ± 42.04	1688.76 ± 53.83	1667.15 ± 54.35	0.837
	CON	1679.50 ± 46.76	1687.07 ± 62.66	1704.35 ± 39.24	0.158
**Protein (g/day)**	HIIT	79.69 ± 6.01	77.69 ± 5.52	79.92 ± 6.06	0.935
	MICT	78.00 ± 5.91	80.76 ± 8.02	77.84 ± 4.65	0.916
	CON	81.71 ± 6.00	81.85 ± 4.80	81.28 ± 4.89	0.833
**carbohydrate (g/day)**	HIIT	206.61 ± 9.91	208.00 ± 9.49	213.30 ± 7.20	0.169
	MICT	208.61 ± 5.33	206.76 ± 7.72	247.73 ± 6.00	0.796
	CON	206.85 ± 7.22	20.8.28 ± 9.65	214.92 ± 9.36	0.117
**Fat (g/day)**	HIIT	59.00 ± 3.55	58.84 ± 3.43	56.53 ± 4.33	0.133
	MICT	58.30 ± 2.59	59.84 ± 4.14	57.61 ± 3.01	0.528
	CON	58.35 ± 3.22	58.50 ± 3.77	58.71 ± 3.47	0.758

**Abbreviation: HIIT**, High Intensity Interval Training; **MICT**, Moderate-Intensity Continuous Training; **CON**, Control; Values are Mean ± SD; Significant values are set at *p* < 0.05.

**Table 3:** Effects of group on post values controlling pre values using ANCOVA and Bonferroni’s multiple comparison test.

Body Composition and Physical Fitness					Inflammatory Markers				
Variable	Contrast	β (SE)	95% CI	*p* Value *	Variable	Contrast	β (SE)	95% CI	*p* Value *
BM-Post (kg)	HIIT vs. MICT	−0.92 (0.25)	−1.54, −0.30	0.002	IL-8-Post (pg/mL)	HIIT vs. MICT	0.16 (0.44)	−0.96, 1.27	1.000
	HIIT vs. CON	−1.86 (0.24)	−2.46, −1.25	<0.001		HIIT vs. CON	0.05 (0.42)	−1.02, 1.12	1.000
	MICT vs. CON	−0.94 (0.24)	−1.54, −0.33	0.001		MICT vs. CON	−0.11 (0.42)	−1.15, 0.94	1.000
FM-Post (kg)	HIIT vs. MICT	−0.64 (0.11)	−0.92, −0.35	<0.001	IL-10-Post (pg/mL)	HIIT vs. MICT	0.41 (0.39)	−0.58, 1.40	0.925
	HIIT vs. CON	−0.77 (0.11)	−1.05, −0.48	<0.001		HIIT vs. CON	1.02 (0.38)	0.07, 1.98	0.033
	MICT vs. CON	−0.13 (0.12)	−0.42, 0.17	0.859		MICT vs. CON	0.62 (0.38)	−0.35, 1.58	0.357
LM-Post (kg)	HIIT vs. MICT	−0.05 (0.20)	−0.56, 0.46	1.000	IL-6-Post (pg/mL)	HIIT vs. MICT	−0.35 (0.20)	−0.86, 0.16	0.285
	HIIT vs. CON	0.33 (0.20)	−0.17, 0.82	0.310		HIIT vs. CON	−0.61 (0.20)	−1.11, −0.10	0.014
	MICT vs. CON	0.37 (0.20)	−0.13, 0.88	0.210		MICT vs. CON	−0.26 (0.20)	−0.76, 0.25	0.629
VO_2peak_-Post (mL·kg^−1^·min^−1^)	HIIT vs. MICT	0.25 (0.13)	−0.07, 0.58	0.178	TNF-α-Post (pg/mL)	HIIT vs. MICT	−0.94 (0.29)	−1.66, −0.22	0.007
	HIIT vs. CON	0.98 (0.12)	0.67, 1.29	<0.001		HIIT vs. CON	−2.02 (0.28)	−2.72, −1.32	<0.001
	MICT vs. CON	0.73 (0.12)	0.42, 1.03	<0.001		MICT vs. CON	−1.08 (0.27)	−1.76, −0.39	0.001
UBS-Post (kg)	HIIT vs. MICT	−0.01 (0.37)	−0.95, 0.92	1.000	Leptin-Post (ng/mL)	HIIT vs. MICT	−0.19 (0.06)	−0.33, −0.04	0.007
	HIIT vs. CON	0.69 (0.37)	−0.22, 1.61	0.198		HIIT vs. CON	−0.39 (0.06)	−0.53, −0.25	<0.001
	MICT vs. CON	0.71 (0.37)	−0.23, 1.64	0.195		MICT vs. CON	−0.20 (0.06)	−0.34, −0.06	0.004
LBS-Post (kg)	HIIT vs. MICT	1.28 (0.43)	0.20, 2.37	0.015	Adiponectin-Post (ng/mL)	HIIT vs. MICT	0.15 (0.14)	−0.21, 0.51	0.915
	HIIT vs. CON	2.97 (0.42)	1.91, 4.03	<0.001		HIIT vs. CON	0.30 (0.14)	−0.06, 0.67	0.126
	MICT vs. CON	1.69 (0.42)	0.64, 2.74	<0.001		MICT vs. CON	0.15 (0.14)	−0.20, 0.51	0.862

**Abbreviations.** BM, body mass; FM, fat mass; LM, lean mass; UBS, upper body strength; LBS, lower body strength; IL-8, interleukin-8; IL-10, interleukin-10; IL-6, interleukin-6; TNF-α, tumor necrosis factor-alpha. β (the slope of the regression line), In the ANCOVA model is a regression coefficient for the relationship between interventions and post-markers. In addition, β is the amount of impact of interventions on markers in the post-test when adjusting for markers in the pre-test. * The Bonferroni test for multiple comparison.

## Data Availability

Data sharing is applicable with email to corresponding authors.
